# Sierra Salviæ in Remittent Fever

**Published:** 1884-01-20

**Authors:** Francis A. Evans

**Affiliations:** Newbern, Tenn.


### SIERRA SALVIÆ IN REMITTENT FEVER.

BY FRANCIS A. EVANS, M.D., NEWBERN, TENN.

Sometime ago, I saw the idea advanced that mountain sage (artemisia frigida) or,as it is pleasantly called in the Spanish tongue, "sierra salvia," was an excellent substitute for quinine; and as a substitute, possessing positive power for quinine, would be very valuable, indeed, I determined, having secured some from Parke, Davis & Co., the enterprising chemists, to give it a thorough trial. I am now very well satisfied that it is a remedy possessing pronounced anti-malarial qualities, similar to the cinchona bark and preparations; and I wish to report three cases, taken from several, in which I used it successfully:

CASE I.—I was called, October 2d, to see R. B——, æt. about 40, living near Onion River. Found patient with high fever, tongue broad and coated dirty whitish yellow, pulse full and bounding, constipated bowels, severe headache, pains in back, vomiting occasionally, morning remissions, etc. Veratrum vir. tinc. in two drop doses, every half hour, was administerad, until abatement of temperature, then quinine. The quinine was promptly rejected by the stomach. I then ordered:

℞ Tr. veratrum .................................. gtts. xx,
Fl.. ext. sierra salviæ .......................... ʒ ij,
Aqua ad .......................................... oz. iv.
M. Sig. Teaspoonful every two hours.

Saw patient next day and found him much better. Continued treatment.

Saw him on 5th, still improving. Ordered soups, etc. Left powder to move the bowels.

Saw patient again on the 7th; found him sitting up. Tongue clean; appetite good. Dismissed him.

CASE II.—Precisely like case one. Treatment and results same.

CASE III. Mrs. H., æt. about 30. Had premonitory symptoms of ordinary remittent fever for two days, when a pronounced attack set in. I was called twenty-four hours later. Found her delirious; pulse small, but tolerably soft; face flushed; eyes bright, with slightly contracted pupils; bowels regular; tongue narrow, elongated, with edges very red; great tenderness over region of stomach, occasionally vomiting. Ordered:

℞ Fl. ext. aconite ................................ gtt. xxx,

Fl. ext. gelsemium ................................ ʒ j,

Aqua ad .......................................... oz. iv.

M. Sig. Teaspoonful every two hours, alternating with,

℞ Acidi hydrochlor. dilut..............................ʒ ss,

Syrup simplex..............................oz. iij.

M. Sig. Sip *ad lib.*

Saw the patient next day, and found conditions better. Treatment continued.

Saw patient again the next day; pulse of better volume, still accelerated; tongue decidedly changed for the better. A good morning remission.

Saw her again next day, in evening; pulse 90, moderately soft; tongue normal; appetite returning. Acid treatment stopped. Sedatives continued, with two five gr. Dover's powders during the night. Left the following formula, for to begin next morning early:

℞ Fl. ext. sierra salviæ ........................ʒ ij,

Tr. nux vomica..............................gtt. xv,

Aqua ad..............................oz. iv.

M. Sig. Teaspoonful every two hours.

Saw patient two days later; found her sitting up; clear of fever; appetite fair; bowels slightly constipated. Dismissed her.

I submit these cases for what they are worth, not claiming any originality in the use of the drug. I wish to say in this connection that the remedy was used where I earnestly thought quinine was inadmissible.—*Nash. Jr. Med. and Surg'y.*

